# Enhancing West African family medicine curriculum through entrustable professional activities

**DOI:** 10.4102/phcfm.v16i1.4691

**Published:** 2024-12-13

**Authors:** Bolatito B. Fatusin, Musa Dankyau, Akye Essuman, Abraham N. Gyuse, Akinfemi J. Fatusin, Louis S. Jenkins

**Affiliations:** 1 Department of Family Medicine, Federal Medical Centre, Abeokuta, Nigeria; 2 Department of Family Medicine, Faculty of Clinical Sciences, Bingham University, Karu, Nigeria; 3 Department of Family Medicine, Bingham University Teaching Hospital, Jos, Nigeria; 4 Department of Internal Medicine, School of Medicine, University of Health and Allied Sciences, Ho, Ghana; 5 Family Health Centre, Ho Teaching Hospital, Ho, Ghana; 6 Department of Family Medicine, Faculty of Clinical Sciences, University of Calabar, Calabar, Nigeria; 7 Chief Tony Anenih Geriatric Centre, University College Hospital, Ibadan, Nigeria; 8 Department of Family and Emergency Medicine, Faculty of Medicine and Health Sciences, Stellenbosch University, Cape Town, South Africa; 9 Primary Health Care Directorate, Department of Family, Community and Emergency Care, Faculty of Medicine and Health Sciences, University of Cape Town, Cape Town, South Africa; 10 Department of Family and Emergency Medicine, George Hospital, George, South Africa

**Keywords:** curriculum, family medicine, medical education, workplace based assessment, West Africa, primary care, entrustable professional activities

## Abstract

The training of Family Medicine residents in the West Africa College of Physicians (WACP) has steadily upscaled to a competency-based approach over the years. The latest review of the curriculum (2022) includes self-directed online modules on clinical postings, health management, patient safety, quality assurance research and medical education among others. The operationalisation of the revised curriculum involves the use of workplace-based tools for formative assessments. However, some shortcomings of the traditional work place based assessment (WPBA) have been observed, including a lack of standardisation, time consumption, variability in the assessors’ judgements and systematic biases. These shortcomings can be mitigated through the adoption of entrustable professional activities (EPA) along with the use of WPBA.

## Introduction

The training of Family Medicine residents in the West Africa College of Physicians (WACP) has evolved over the years. Since the first curriculum, which was developed from the already existing curriculum of National Postgraduate Medical College (NPMC) in 1987, it has been reviewed every 5 years.^1^ Each revised edition showed a steady move towards a more competency-based approach to training. The latest review of the curriculum (2022) includes self-directed online modules on clinical postings, health management, patient safety, quality assurance research and medical education.^2^ Other new features include a portfolio of learning, guidelines for writing patient management reports, research proposals, dissertations and competencies required in special interest areas.^2^ The minimum period for training at the college membership stage was extended from 24 to 30 months because of these new inclusions. Likewise, for the fellowship stage, the minimum training period has also been extended from 24 to 30 months.^2^ The operationalisation of the revised curriculum involves the use of workplace-based tools for formative assessments. Summative assessments modalities include: Multiple Choice Questions, Problem Solving Question, Objective Structured Clinical Examination, defence of patient management report and Oral Examination at Membership stage while the defence of portfolio of learning and defence of dissertation occur at the fellowship stage. Although the implementation of the curriculum is still in an early stage making it difficult to assess its impact on training yet, it promises a brighter future for Family Medicine postgraduate training in the West Africa sub-region. To ensure that this new curriculum continues to keep up with the international standard of post-medical graduate training, this opinion article is advocating for the need to consider the inclusion of entrustable professional activities (EPAs) in its subsequent editions.

## Workplace-based assessment

The application of workplace-based assessment (WPBA) in postgraduate medical training is a paradigm shift from the traditional knowledge-based proxy assessment to an outcome-based authentic evaluation.^3^ It entails assessing residents’ progressive development over time of abilities required to practice safely and independently through a combination of observations in clinical practice, supervisor feedback and resident reflections.^4^ These learning events are assessed through the use of WPBA tools such as the Mini Clinical Evaluation Exercise (Mini-CEX) tool, the Direct Observation of Procedural Skills (DOPS) tool, the Clinical Encounter Card (CEC), the Acute Care Assessment Tool (ACAT) and the Multisource Feedback (MSF) tool, among others^5^ Over time, the resident captures these learning events in a learning portfolio, by way of completed tools (forms) and reflections. While many countries across the world now use WPBA in their postgraduate training programmes,^6^ very few programmes in Africa have embarked on implementing WPBA in postgraduate family medicine training. For example, South Africa postgraduate family medicine programme formative assessment is based on WPBA.^7^

The WACP Faculty of Family Medicine has adopted the use of some WPBA tools in the 2022 curriculum.^2^ These include the use of case-based discussions (CbD), DOPS, Mini-CEX, MSF and evaluation of clinical events (ECE) for formative assessments of postgraduate residents. As an educational framework, WPBA has a high content validity based on its ability to assess residents’ actual performance in the workplace.^8^ It facilitates assessment of learning and assessment for learning by integrating teaching, resident learning, summative assessments and formative feedback. Multiple, ‘low stakes’, *ad hoc* WPBA complement the traditional ‘high stakes’ end-of-training period summative assessments. At present, these summative assessments can only address assessment of knowledge, application of knowledge, and performance through picture tests, multiple extended questions and objective structured clinical examinations.^9^

However, some shortcomings of the conventional WPBA have been recorded in the literature, including the lack of standardisation, time consumption, variability in the assessors’ judgements and systematic biases.^10^ Work place based assessment is usually case-specific for each resident assessed. This means clinical workplace performance will vary from one case to the other and from one clinical situation to another. In addition, assessors may differ in their judgements in the presence of similar evidence. This, however, could provide a richer overall picture of performance if assessors focus on different aspects of performance and value different things as indicators of good care. Providing narrative descriptions of the resident’s performance may therefore be more helpful because they are easier for residents to understand and may be interpreted more consistently by other assessors. Furthermore, narrative descriptions allow other assessors and the resident to understand what the experience of the assessor is with regard to the resident’s performance. The importance of narrative feedback in WPBA cannot be overemphasised. Furthermore, having multiple assessors and encouraging them to document their narrative feedback immediately could mitigate saliency biases.^4^ This occurs when the assessor tends to focus on the most prominent skills and abilities of the resident to the detriment of other relevant details. Assessors may also face some dilemmas, between the allocation of time for patient care and observing a resident’s performance, documenting their findings, and providing feedback to residents. As WPBA evolved in medical education, the concept of EPAs was introduced to augment a competency-based curriculum. The EPA brings to fore the essence of adopting WPBA in formative assessment as it identifies specific, high stake tasks that require demonstration of competence. It encourages narrative descriptions of performance and reduces saliency bias.

## Introducing entrustable professional activities

Entrustable professional activities are defined as units of professional practice that encompass one or more tasks that can be entrusted to a trainee or junior doctor once they have demonstrated competence.^11^ They are designed to be observable and measurable. The EPAs define practice expectancies and provide clear learning direction and explicit teaching assessment goals.

The inclusion of EPAs allows for more effective operationalisation of WPBA. While the traditional WPBA may focus on isolated knowledge and skills, neglecting the essential aspect of clinical reasoning within the context of real patients, adopting the EPA approach in medical education requires the integration of several competencies, which makes it easier to assess a resident on a combination of multiple competencies, attitudes, skills and professional ethics.^12^ Furthermore, unlike traditional WPBAs, EPAs make the clinical context explicit, which influences the assessments of residents that might contribute to a good or bad performance. Entrustable professional activity -oriented WPBA takes a broader approach by evaluating residents’ ability to integrate knowledge, analyse patient data and make sound clinical decisions in a real-world setting.

Development of an EPA-based WPBA programme usually involves a long process of review of the assessment processes in the curriculum by a body of experts and careful grouping of different units of competencies that define the professional activities of a specialist. This could take years before it is completed. However, the process can be fast-tracked by adapting the templates used by the countries that have successfully developed EPAs for their family medicine postgraduate programmes, considering its suitability in their context. In the United States (US), 76 EPAs were developed for ambulatory family medicine practice over 3 years by 22 experts and further refined using a Delphi Process.^11^ Likewise, in Canada, experts from four universities designed 35 EPAs in nine curricular domains for the Canadian family medicine residency programme.^13,14^ Recently, the South African Academy of Family Medicine also developed EPAs for postgraduate family medicine programmes.^15^ A national working committee, which comprised representatives of the nine postgraduate training programmes, worked on the project from 2020 to 2022. Subsequently, through a consensus review process, a final list of 22 EPAs was agreed on and incorporated into the WPBA in 2024.^7^ Presently, implementation, user buy-in, adaptation to the revised portfolio of learning and incorporation into the existing curriculum is ongoing.^15^

According to Dr ten Cate from University of Utrecht,^16^ EPAs are defined based on the following criteria: They must be part of the essential professional work of the specialty and not general medical ability; they must require adequate knowledge, skills, and attitudes; they must lead to recognised performance that is unique to a medical doctor; they should be unique to physicians in that specialty; they should be independently executable; they should be executable within a time frame; they should be observable and measurable in its process and outcome. For example, an EPA describing the ability to perform common surgical skills such as appendectomy, herniorrhaphy and excisional biopsy will entail history taking, doing a physical examination, making a diagnosis and implementing a plan of care that is evidence-based and takes into account the needs and values of the patient and the health team. Multiple assessors gather information through various WPBA tools on the resident’s activities at the workplace, which are documented in the learning portfolio. Each learning encounter with the resident represents an ‘*ad hoc*’ low stakes assessment of practice, which are captured as ‘data points’ in the portfolio. The local competency committee, consisting of senior faculty, meets twice a year and reviews the overall data points in every resident’s portfolio to make a high stakes summative assessment of that resident. This allows the resident to progress to the next stage of training. These data points may include various observations, learning plans, resident reflections, supervisors’ feedback, entrustment-based discussions and multi-source feedback.

## Proposed framework for implementing entrustable professional activities in West African Family Medicine

Drawing insights from the literature and the South African experience, a framework for designing an EPA-based WPBA for West African Family Medicine is suggested further in the text:

- **Form a regional EPA working group:**
  ■ Initiate discussions on EPAs during the annual scientific conference meeting and selection of working group members. Members should be representative of the member countries who were involved in the drafting of the 2022 curriculum.
- **Organise EPA training workshops for the committee members:**
  ■ Contacts can be made with the South African family medicine EPA working group chairperson. Set up online and/or face-to-face workshops and sharing of experience.
- **Decide on the need for and feasibility of EPAs in the West African family medicine context:**
  ■ The working group should recommend to the college faculty the need for EPAs and the right timing to introduce the concept into the curriculum. The college faculty should communicate the decision to the family medicine trainers and residents. With faculty resource constraints it is paramount that EPAs are introduced in a very feasible and sustainable manner. For example, decide to use only a few WPBA tools, have at least one learning event captured weekly and enable a learning environment in the clinical workplace.
- **Identify and develop key EPAs:**
  ■ Once consensus has been reached, the working group should review the curriculum to identify and develop key EPAs essential for family medicine residents to master. This can be done through a literature review, expert consensus and focus groups with residents and trainers. Existing EPAs from other family medicine training programmes could serve as possible templates for a starting point.
- **Choose appropriate assessment tools:**
  ■ Once the key EPAs have been identified, the EPAs should be mapped to the appropriate WPBA assessment tools. These tools are already included in the 2022 curriculum.
- **Training of trainers (TOT):**
  ■ All family medicine consultants involved in the training of residents should be trained on the EPAs and the assessment tools. Training should focus on the principles of EPA-based assessment, as well as the specific tools that will be used. Giving effective feedback is particularly important. Residents should receive regular feedback on their performance of the EPAs. This feedback should be specific, timely and actionable.
- **Implement EPA directed WPBA:**
  ■ The EPAs and assessment tools should be integrated into a programmatic WPBA process. Residents should be given opportunities to demonstrate their competence in performing the EPAs through various clinical experiences. Evidence of learning and assessments should be captured in a portfolio of learning.
- **Modification of EPAs:**
  ■ The EPAs should be revisited and revised after a period of implementation. Subsequent revision of the curriculum should update the EPAs based on feedback.

## Benefits of Implementing entrustable professional activities

Adopting EPAs for WPBA in the revised WACP Family Medicine Curriculum should offer several benefits, which include:

- **Standardised Assessment:** The EPAs provide a shared understanding of expected performance standards.
- **Clinical Relevance:** The EPAs are based on real-world tasks that family medicine residents are expected to perform, ensuring that the assessment is relevant to their future practice.
- **Focus on Competence:** The EPAs emphasise the assessment of clinical competence rather than knowledge alone, which aligns with the goals of the revised WACP curriculum, therefore requiring design of appropriate WPBA opportunities.
- **Empowerment of Residents:** The EPAs provide residents with a clear understanding of curriculum expectations and help them to track their progress towards achieving competence.
- **Aligning with International benchmarking of postgraduate training:** It should enable easier comparison of graduates across programmes and even countries if similar EPAs are used.
- **Attainment of the goal of the training programme:** The EPAs will help achieve the goals of postgraduate family medicine training, which is to produce Family Medicine specialists of international standard who can meet the peculiar health needs of the West African people.
- **Scalability:** The EPAs can be adapted to different levels of training, from novice to expert, making them scalable across the entire educational continuum.
- **Supports formative feedback:** The EPAs support a continuum of learning by providing formative feedback that guides learners from supervised practice to independent performance.

## Conclusion

The adoption of EPAs for WPBA in the revised WACP Family Medicine Curriculum has the potential to improve the quality and effectiveness of residents’ assessment. By providing a structured framework that focuses on competence and clinical relevance, EPAs can help to ensure that residents are well-prepared for the practice of family medicine. When the 2022 curriculum is reviewed, the inclusion of EPA should be considered in WPBA by the WACP Faculty of Family Medicine to further enhance its effectiveness. Lessons learnt from this process could be useful to other training programmes in Africa or similar contexts globally.
